# Effect of different antioxidants in ischemia-reperfusion syndrome

**DOI:** 10.1186/cc9516

**Published:** 2011-03-11

**Authors:** W Hoyos, L Alfaro, B Garcia-Prieto, G Lopez, P Flores

**Affiliations:** 1 Universidad, Santa Tecla, La Libertad, El Salvador

## Introduction

The ischemia-reperfusion syndrome commonly seen in different clinical scenarios leads to acute renal failure and it is known that the free oxygen radicals play an important role in the pathophysiology of this injury. Recent studies suggest that the use of antioxidants can provide renal protection, reducing parenchymal lesions and expression of inflammatory mediators, improving renal function, resulting in a better outcome.

## Methods

We studied the effect of DMSO, DMSO-ascorbic acid and DMSO-*N*-acetylcysteine administration on renal injury induced by I/R. Thirty minutes renal ischemia was induced in 50 male, New Zealand rabbits. The subjects were divided into five groups: (A) Sham, unilateral nephrectomy, no ischemia induced. (B) Control group. (C) DMSO, unilateral nephrectomy, I/R treated with DMSO 3.8 mg/kg. (D) DMSO-ascorbic acid, unilateral nephrectomy, I/R treated with ascorbic acid 150 mg/kg and DMSO 3.8 mg/kg. (E) DMSO-*N*-acetylcysteine unilateral nephrectomy, I/R treated with *N*-acetylcysteine 20 mg/kg and DMSO 3.8 mg/kg. All subjects were given 8 hours of reperfusion. Two blood samples were taken at baseline and after the reperfusion phase. Each sample was tested for serum creatinine. After reperfusion left nephrectomy was performed on each subject before euthanasia. A pathological analysis evaluated tubular and basement membrane changes. The level of injury was scaled in three stages: mild, moderate and severe.

## Results

**Figure 1:**
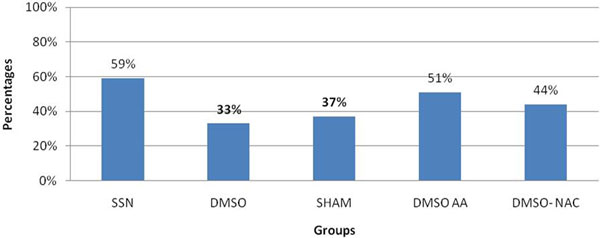
**Total histopathological renal damage**.

**Figure 2:**
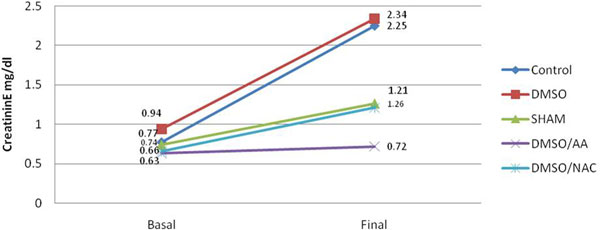
**Mean serum creatinine values in the different groups**.

The histological analysis showed a total damage in 59% of the control group, compared with DMSO 33%, DMSO-AA 51%, and DMSO-NAC 44% (Figure 1). Also, inflammatory properties were absent or to a lesser extent in those groups who used antioxidants. Serum creatinine analysis in the control group showed higher values compared with the association of DMSO-AA, DMSO-NAC where the increases were lower (Figure 2).

## Conclusions

The findings imply that reactive oxygen species play a causal role in I/R-induced renal injury, and that antioxidants exert renoprotective effects, probably by radical scavenging and antioxidant activities, in this way diminishing renal function deterioration.
